# Entrustable professional activities-based objective structured clinical examinations in a pharmacy curriculum

**DOI:** 10.1186/s12909-024-05425-y

**Published:** 2024-04-22

**Authors:** Soumana C. Nasser, Roy Kanbar, Imad F. Btaiche, Hanine Mansour, Reine Elkhoury, Carl Aoun, Lamis R. Karaoui

**Affiliations:** 1 Pharmacy Practice Department, School of Pharmacy, Lebanese American University, Blat, P.O. Box 36, Byblos, Lebanon; 2 Pharmaceutical Sciences Department, School of Pharmacy, Lebanese American University, Blat, P.O. Box 36, Byblos, Lebanon; 3 School of Pharmacy, Lebanese American University, Blat, P.O. Box 36, Byblos, Lebanon

**Keywords:** Entrustable professional activities, Objective structured clinical examinations, Pharmacy education, Competency assessment, Simulation, Quality education

## Abstract

**Background:**

The integration of Objective Structured Clinical Examinations (OSCEs) within the professional pharmacy program, contributes to assessing the readiness of pharmacy students for Advanced Pharmacy Practice Experiences (APPEs) and real-world practice.

**Methods:**

In a study conducted at an Accreditation Council for Pharmacy Education (ACPE)-accredited Doctor of Pharmacy professional degree program, 69 students in their second professional year (P2) were engaged in OSCEs. These comprised 3 stations: best possible medication history, patient education, and healthcare provider communication. These stations were aligned with Entrustable Professional Activities (EPAs) and Ability Statements (AS). The assessment aimed to evaluate pharmacy students’ competencies in key areas such as ethical and legal behaviors, general communication skills, and interprofessional collaboration.

**Results:**

The formulation of the OSCE stations highlighted the importance of aligning the learning objectives of the different stations with EPAs and AS. The evaluation of students’ ethical and legal behaviors, the interprofessional general communication, and collaboration showed average scores of 82.6%, 88.3%, 89.3%, respectively. Student performance on communication-related statements exceeded 80% in all 3 stations. A significant difference (*p* < 0.0001) was found between the scores of the observer and the SP evaluator in stations 1 and 2 while comparable results (*p* = 0.426) were shown between the observer and the HCP evaluator in station 3. Additionally, a discrepancy among the observers’ assessments was detected across the 3 stations. The study shed light on challenges encountered during OSCEs implementation, including faculty involvement, resource constraints, and the necessity for consistent evaluation criteria.

**Conclusions:**

This study highlights the importance of refining OSCEs to align with EPAs and AS, ensuring a reliable assessment of pharmacy students’ clinical competencies and their preparedness for professional practice. It emphasizes the ongoing efforts needed to enhance the structure, content, and delivery of OSCEs in pharmacy education. The findings serve as a catalyst for addressing identified challenges and advancing the effectiveness of OSCEs in accurately evaluating students’ clinical readiness.

**Supplementary Information:**

The online version contains supplementary material available at 10.1186/s12909-024-05425-y.

## Background

The Objective Structured Clinical Examinations (OSCEs) were first described by Harden et al. in 1975 with their purpose to objectively assess the clinical competence of medical students in a safe practice environment using standardized medical case scenarios that simulate real case scenarios [1]. In 1988, Harden further refined the definition of OSCEs as “an approach to the assessment of clinical competence in which the components of competence are assessed in a planned or structured way with the attention being paid to the objectivity of the examination” [2]. Compared to other assessments of learning in practice, OSCEs are generally more objective and less biased considering the input of several examiners along with the use of different evaluation rubrics in the process [3].

Historically, pharmacy programs have mostly relied on standardized exams in the formative assessment of students’ learning outcomes. However, the shift of pharmacy education from the product to the patient has required the adoption of competency-based assessments such as OSCEs that objectively appraise students’ hands-on clinical competence, critical thinking, teamwork, problem-solving and communication skills, among other required competencies in patient care [4, 5]. Accordingly, OSCEs are being increasingly used by pharmacy programs as formative and summative assessments of students’ application of knowledge and their readiness for practice and team work [6, 7]. Furthermore, OSCEs have become part of the licensing examination for pharmacists in Canada and have been recommended for inclusion in the competency-based learning and assessment by several societies and accreditation agencies [4, 8].

Standard 24 of the 2016 accreditation standards of the Accreditation Council for Pharmacy Education (ACPE) requires that “the college or school develops, resources, and implements a plan to assess attainment of educational outcomes to ensure that graduates are prepared to enter practice” [9]. ACPE provides guidance documents for the competencies and skills that students should demonstrate at each level of knowledge including the domains and Ability Statements (AS) that are central to the preparation of pharmacy students prior to their advanced pharmacy practice experiences (APPEs) [10]. In addition, the core Entrustable Professional Activities (EPAs), published by the American Association of Colleges of Pharmacy (AACP), define the “essential activities and tasks that all new pharmacy graduates must be able to perform without direct supervision upon entering practice” [11]. AS and EPAs are matched with the educational outcomes of the Center of Advancement of Pharmacy Education (CAPE) 2013 and the Pharmacists’ Patient Care Process (PPCP) [12]. While both AS and EPAs share a common objective of ensuring that pharmacy graduates are prepared for practice, they differ however in their approach and focus. AS delineate the knowledge, skills, attitudes and abilities expected from pharmacy graduates and typically serve as a guide for curriculum development. EPAs, however, are specifically focused on the practical tasks and responsibilities that pharmacy graduates should be entrusted to perform autonomously and serve as the basis for competency assessment in evaluating students’ work-readiness.

Despite the widespread acceptance of EPAs in pharmacy education, their integration in learning and assessment practices remains limited and inconsistent [13]. For instance, it is argued that EPAs are professional responsibilities and should not be used to assess performance in a classroom setting because such assessment requires direct and multiple observations of the learner performing the EPA without supervision [14]. Yet, there are reports of EPAs being successfully implemented in experiential pharmacy settings and OSCEs [15–17]. Accordingly, EPAs were reported as reliable assessment tools to assess pharmacy students’ performance in their first professional year as part of introductory pharmacy practice experiences (IPPEs), in the first pharmacy professional year, or in OSCEs [16–17]. Yet, there remains a need to evaluate the use of EPAs in later stages of pharmacy education particularly nearing graduation [18].

Accordingly, the aim of this study was to describe the development and implementation of a pharmacy OSCE with a focus on EPAs and AS, and report the student performance on competencies related to APPE- and practice-readiness such as ethical and legal behaviors, general communication skills, and interprofessional collaboration.

## Methods

### Description of the educational activity and setting

The Doctor of Pharmacy (Pharm.D.) program at the Lebanese American University (LAU) School of Pharmacy spans six years, and is accredited by ACPE. It is a 201 credit-hour program including 61 didactic and 15 experiential courses that cover a diverse range of areas including biomedical, pharmaceutical, social, behavioral, administrative, and clinical sciences. Experiential education that comprises students’ internships in hospitals, community pharmacies and other healthcare settings is integrated throughout the program. Successful completion of all the required courses is mandatory for graduation.

In 2019, a school-based OSCEs workgroup implemented a plan for longitudinal simulation activities in professional years 1 (P1), 2 (P2) and 3 (P3). The OSCEs blueprint is based on the core EPAs of AACP and the pre-APPE Performance Domains and Abilities of the 2016 ACPE standards guidance document [8–10]. All OSCEs were conducted in LAU’s Clinical Simulation Center. The cohort included all student pharmacists with a P2 standing who are qualified to progress into P3 i.e., carrying no more than one P2 didactic course to P3. The LAU SOP OSCEs is not yet a high-stakes exam. However, if a student misses or does not pass the OSCE, they will sit for a make-up OSCE.

There were 3 different OSCE stations: station 1 for best possible medication history; station 2 for patient education; and station 3 for healthcare provider (HCP) communication. Station 1 focused on eliciting comprehensive medical and medication histories that are essential elements of patient care. Students are engaged with a simulated scenario involving a patient admitted to the emergency department with severe headache, tasked with gathering pertinent information to guide subsequent clinical decisions. At Station 2, students are tasked with patient education, particularly counseling on the appropriate usage of anticoagulant medication, while fostering patient understanding and compliance [see supplementary material 1]. Station 3 is dedicated to refining communication with healthcare providers (HCPs) and practicing clinical decision-making within a collaborative framework. In this station, students reviewed a patient chart, reconciled the discharge medications, and engaged in dialogue with physicians to propose and discuss recommended interventions. This station underscores the importance of effective interprofessional communication and teamwork in optimizing patient outcomes.

OSCEs stations were mapped to the AS (Table 1) and EPAs (Table 2). The 3 stations were found to map to 6 out of the 11 AS and to 8 out of the 15 EPAs. Specifically, station 1 addressed AS2 (basic patient assessment) and EPA1 (collect patient information). Station 2 focused on AS8 (patient education) and EPA11 (patient and HCP education). Station 3 addressed AS7 (general communication abilities) and EPA6 (interprofessional collaboration). Of note, AS6 (ethics, professional, and legal behavior), AS7 and EPA 6 were assessed in all 3 stations. AS pertaining to patient safety, pharmaceutical calculations, drug information analysis, health and wellness, and insurance/prescription drug coverage were not appropriately aligned with the competencies evaluated in the stations and were consequently not assessed further in this study. Similarly, the EPAs associated with identifying patients at risk for prevalent diseases in a population, maximizing the appropriate use of medications, ensuring patient immunization against vaccine-preventable diseases, utilizing evidence-based information for patient care advancement, overseeing pharmacy operations for a designated work shift, fulfilling medication orders, and crafting a written plan for continuous professional development, were not aligned with the competencies evaluated in the stations. Consequently, these EPAs were excluded from further assessment in this study.

**Table 1:** Mapping of objective structured clinical examinations (OSCEs) stations to ability statements (AS)

Ability Statements (AS*)	Station 1: Best Possible Medication History	Station 2: Patient Education	Station 3: Health Care Professional
AS2Basic patient assessment	X (main goal)	X	
AS3Medication information		X	X
AS4Identification, assessment, and resolution of drug-related problems		X	
AS6Ethical, professional, and legal behavior.	X	X	X
AS7General communication abilities	X	X	X (main goal)
AS8Patient education		X (main goal)	

* Not covered: AS1 (patient safety), AS5 (mathematics), AS9 (drug information analysis and literature research), AS10 (health and wellness), and AS11 (insurance/prescription drug coverage)

**Table 2:** Mapping of objective structured clinical examinations (OSCEs) stations to the entrustable professional activities (EPAs)

Entrustable Professional Activities	Station 1: Best Possible Medication History	Station 2: Patient Education	Station 3: Health Care Professional
EPA1Collect information to identify a patient’s medication-related problems and health- related needs	X (main goal)	X	
EPA2Analyze information to determine the effects of medication therapy, identify medication-related problems, and prioritize health- related needs		X	X
EPA3Establish patient-centered goals and create a care plan for a patient in collaboration with the patient, caregivers, and other health professionals that is evidence-based and cost-effective	X		X
EPA4Implement a care plan in collaboration with the patient, caregivers, and other health professionals		X	
EPA5Follow-up and monitor a care plan		X	X
EPA6Collaborate as a member of an interprofessional team	X	X	X (main goal)
EPA8Minimize adverse drug events and medication errors		X	
EPA11Educate patients and professional colleagues regarding the appropriate use of medications		X (main goal)	X

* Not covered EPAs: EPA7 (identify patients at risk for prevalent diseases in a population), EPA9 (maximize the appropriate use of medications), EPA10 (in a population, ensure that patients have been immunized against vaccine-preventable diseases), EPA12 (use evidence-based information to advance patient care), EPA13 (oversee the pharmacy operations for an assigned work shift), EPA14 (fulfill a medication order), and EPA15 (create a written plan for continuous professional development)

Each station was mapped to a major AS and EPA in consultation with faculty content experts based on AS and EPA guiding documents. For each station, students interacted with a standardized patient (SP) and/or an HCP. In each station, assessment was performed by a faculty observer and other evaluators, either SP or HCP, depending on the activities and nature of the station. Debriefing was thereafter performed in small groups, during which students shared their feedback on the strengths and weaknesses of their experience and suggested areas for improvement.

Students were evaluated by a faculty member who was present in each station as an observer, in addition to the SP in stations 1 and 2 and the standardized HCP in station 3. The HCP is best portrayed by a faculty member who is well-versed in medical terms for optimal interaction, considering that this is the only station where the discussion is not conducted with a patient. The observer was considered an objective evaluator who filled the checklist for the interaction between the HCP and the student, similar to other stations. Grading rubrics and checklists were used to assess students’ clinical competencies, communication skills and attitude. Checklists for stations 1 and 2 were completed by the faculty observer and the SP evaluator, while the station 3 checklist was completed by the faculty observer and the HCP evaluator. Thus, students’ performance was assessed using two evaluation forms completed for each student per station. In addition, students’ scores per AS and EPA were computed as a direct assessment method to evaluate students’ achievements and their readiness for APPEs and practice, respectively.

Each station is modeled to mimic a real clinic setting and has a duration of 15 min. A coordinator is assigned to proctor and oversee the timing. Students are provided with a guide map that outlines the sequence of the 3 designated stations, to be completed consecutively within a 45-minute timeframe. Every student rotates through an identical set of 3 stations, each featuring the exact same scenarios. Students are directed to spend up to 4 min reading the initial scenario, which is placed on a table outside the station. This scenario is accompanied by a concise patient chart and a brief outline of the tasks that the student must complete during the subsequent 10-minute interaction inside the station. During this interaction, students engage with either a SP or HCP, depending on the theme of the station. If time allows, the evaluator may offer feedback to the student while waiting before exiting the station at the end of the 15 min. Prior to the cumulative OSCEs, faculty delivered orientation sessions to both students and SPs along with generic checklists and also engaged them in mock OSCEs to simulate scenarios in preparation for the actual assessments. Of note, the SPs are recruited by the LAU Clinical Simulation Center and trained by school faculty in preparation for the pharmacy-based OSCEs.

In preparation for the cumulative OSCEs at the end of P2, students were exposed in formative simulation sessions that are integrated in designated P1 and P2 courses (professional communication, dosage forms, select pharmacotherapeutics, and pharmaceutical care and dispensing). These are aimed at advancing students’ knowledge, clinical skills and professional attitudes including professional communication, disease screening, medication preparation, dose calculation, and patient education and counseling. Students are assessed through grading rubrics and checklists. OSCEs are integrated into the summer experiential education course in the P2 year. The assessment of students’ performance in OSCEs were included as an evaluation component of this course.

### Statistical methods

To report student performance on competencies related to the APPE and practice readiness, data were collected during OSCEs, then entered and tabulated in an Excel spreadsheet while maintaining anonymity, and analyzed using SPSS® v27, 2022. The evaluation rubrics and checklists were mapped to EPAs and AS. Grades were calculated for AS and EPAs. A grade of 70% or higher on an AS and EPAs was considered as passing. Analysis of variance (one-way ANOVA) was used to compare students’ scores between stations and across evaluators. The t-test was used to compare scores between evaluators (SP and HCP). All values are expressed as means. Differences were considered significant when a priori *p* value was lower than 0.05.

The study was approved by the LAU Institutional Review Board and granted exempt status under the code number LAU.SOP.HM2.12/Sep/2023. Access to any identifiable data were limited to the investigators and data documents were stored within locked locations. Security codes were assigned to computerized records with names replaced with codes to ensure that data were analyzed without revealing the identity of the participants.

## Results

**Fig. 1:**
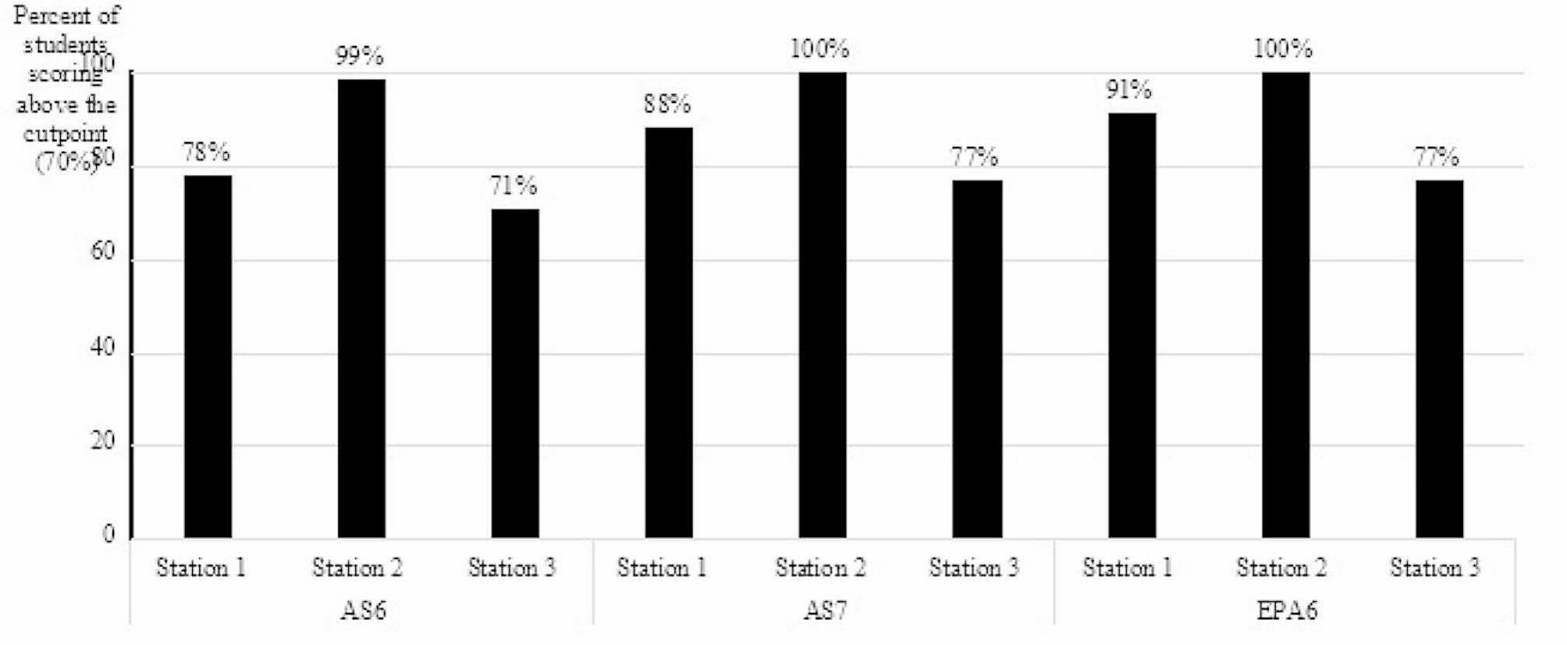
Evaluation of students’ ethical behavior (AS6), interprofessional communication (AS7), and collaboration (EPA6). Percent of students (*N* = 69) achieving the related Ability Statement (AS) and Entrustable Professional Activity (EPA) per station

In total, 69 P2 students completed the three-station OSCEs. The evaluation of ethical and legal behaviors (mapped to AS6), the interprofessional general communication (AS7) and collaboration (EPA6) revealed respectively average scores of 82.6%, 88.3%, 89.3% of students scoring above the 70% cut point across all stations. It is worth noting that the lowest student scores were observed in station 3 where students were evaluated by a HCP (Fig. 1).

**Fig. 2:**
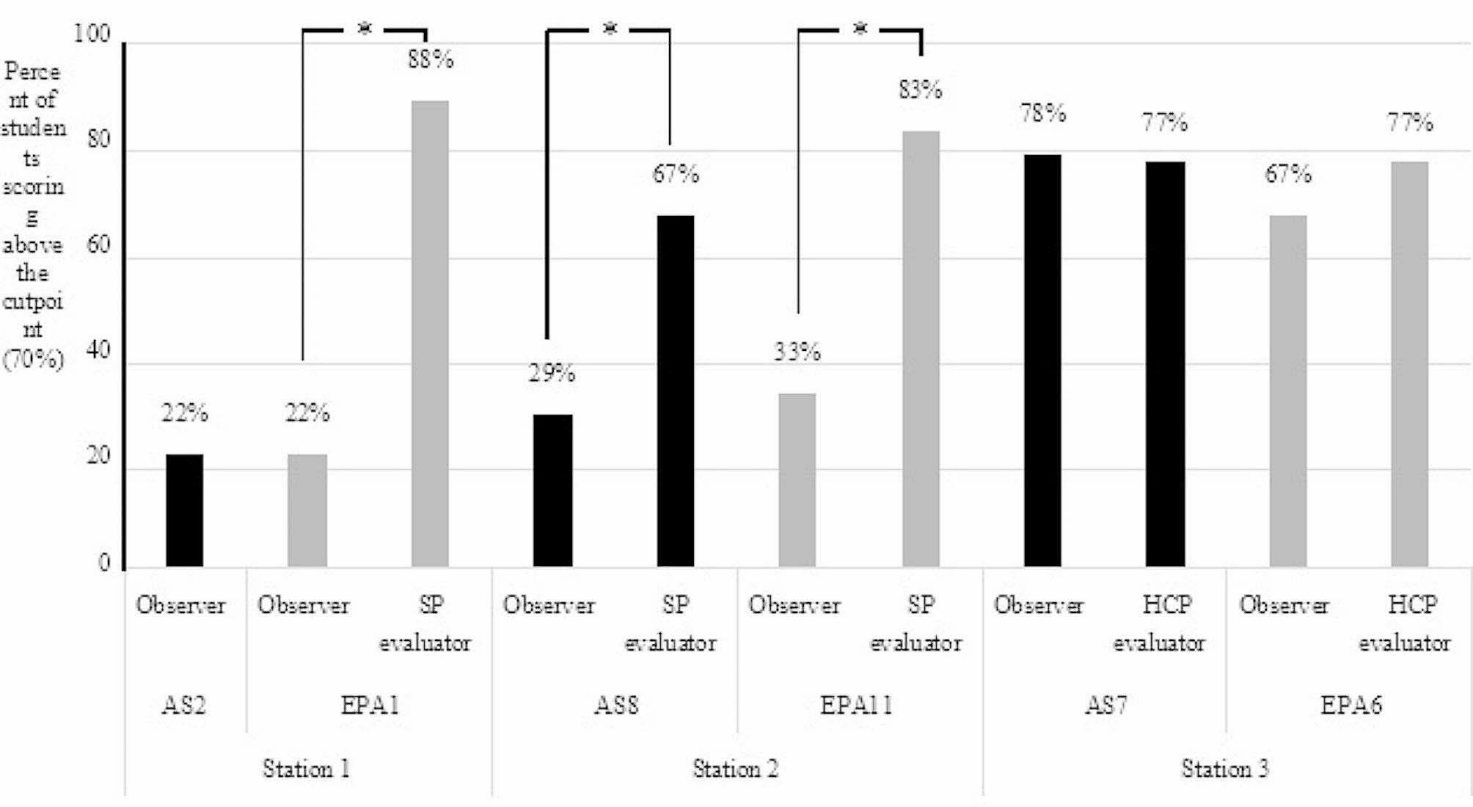
Direct Assessment of APPEs and practice readiness of students per OSCE station. Percent of students (*N* = 69) achieving the related Ability Statement (AS) and Entrustable Professional Activity (EPA) per station (* *p* < 0.0001)

Based on the direct assessment of student APPE- and practice-readiness per station, the observer ratings showed satisfactory ratings were observed in station 3 for AS7 (78%) and EPA6 (67%) as opposed to suboptimal performance in stations 1 and 2 for AS2-basic patient assessment (22%), EPA1-collect patient information (22%), AS8-patient education (29%) and EPA11-patient and HCP education (33%). This indicates a discrepancy in the observer assessments across the 3 stations. A significant difference using t-test (*p* < 0.0001) was detected between the scores of the observer and the SP evaluator in stations 1 (EPA1: observer 22% vs. SP evaluator 88%; EPA 11: observer 33% vs. SP evaluator 83%) and 2 (AS8: observer 29% vs. SP evaluator 67%). Scores of station 3 (*p* = 0.426) showed comparable results between the observer and the HCP evaluator, implying a higher level of agreement in their evaluations (Fig. 2).

The majority of the SP and HCP evaluators’ scores on communication-related statements in each of the OSCE stations exceeded 80% (Table 3).

**Table 3:** Evaluator scores on communication skills in Objective Structured Clinical Examination (OSCE) stations

	Station 1: Best Possible Medication History	Station 2: Patient Education	Station 3: Health Care Professional	p value (*)
*Communication Assessment*				
1. Allowed patient or HCP to speak without interruption	94.93%	95.65%	90.58%	0.3326
2. Started each new topic of information with an open-ended question	89.13%	94.20%	94.20%	0.0725
3. Used smooth and appropriate transitions during encounter	83.33%	89.86%	81.16%	0.6554
4. Used effective pacing during the encounter (no rushing; not too much silence)	87.68%	90.58%	72.46%	0.1835
5. Voiced empathy for patient situation/problem	81.88%	86.96%	32.61%	0.9999
6. Summarized information back when appropriate.	39.13%	70.29%	53.62%	0.9996
7. Elicited questions and concerns	73.19%	97.83%	73.19%	0.9273
8. Served as patient advocate	78.26%	85.51%	55.07%	0.9962
9. Used appropriate terms while communicating	86.96%	89.86%	92.75%	0.0568
*Eliciting Information*				
10. Made frequent eye contact while patient was speaking	98.55%	98.55%	90.58%	0.8034
11. Maintained an appropriate distance during the encounter	92.03%	98.55%	97.10%	0.5783
12. Body language and/or tone of voice communicated caring and concern	86.96%	97.83%	82.61%	0.1863
13. Showed interest (not bored; did not ignore my statements)	89.13%	99.28%	88.41%	0.6166
*Non-verbal Communication*				
14. Evaluator would be willing to recommend a patient to see this student pharmacist	78.99%	84.80%	60.87%	0.9953
*Analytical checklist*				
15. Student pharmacist properly verifies the patient’s (or HCP) identity (using information from the patient (or HCP) profile such as address or date of birth)	91.30%	95.65%	96.38%	0.6643
16. Student pharmacist identifies self by giving her/his name and title	96.38%	100%	96.38%	0.5239
17. Student pharmacist states the purpose for the discussion	97.10%	89.86%	90.58%	0.7568

*One-way ANOVA test is used to compare student scores between stations on each statement. Significant difference if *p* < 0.05

However, the statement “summarized information back when appropriate” received scores below or equal to 70% in all stations, suggesting an area that may require improvement. For the statement “voiced empathy for patient situation/problem,” the scores given by the HCP evaluators in station 3 were significantly lower (32.6%) compared to the scores provided by the SP evaluators in stations 1 (81.8%) and 2 (86.9%) (*p* < 0.00001). Similarly, for the statement “served as a patient advocate,” the HCP scores in station 3 (55%) were significantly lower than the SP scores in stations 1 (78.26%) and 2 (85.51%) (*p* < 0.00001). Furthermore, in the statement “the evaluator would be willing to recommend a patient to see this student pharmacist,” the HCP scores in station 3 (60.87%) were significantly lower than the SP scores in station 1 (78.9%) and 2 (84.8%) (*p* = 0.00014).

The OSCEs coordinator received suggestions during the debriefing sessions. Those included: adjusting the allocated time for each station based on the task, simplifying the case scenario to enhance focus on the main station goal, clearly highlighting the theme of each station on the opening scenario at the entrance of each station, integrating role play into the preparatory orientation for each station, and ensuring key information is included on both the grading checklist and the patient case scenario.

## Discussion

OSCEs have become more commonly utilized in pharmacy programs as a way to assess students’ practice competencies and as a capstone exam at the end of P2 to assess students’ APPE-readiness [19]. Moreover, OSCEs have been aligned with pharmacy curricula [20], pharmacotherapeutics courses [21], program educational outcomes to assess APPE-readiness [19], and general pharmacy practice competencies such as critical thinking, patient care process and communication skills [22–23].

Since the release of the report on the core EPAs by the 2015–2016 AACP Academic Affairs Standing Committee of AACP, EPAs have been considered as a new approach to define and assess pharmacy practice skills that mark a shift from time-based to outcome-based learning [11, 24, 25]. EPAs are expected to be tasks that are observable, measurable, and require professional training for execution [26]. Various applications of EPAs have been proposed, yet they all converge to prepare graduates for professional practice [26–29] and to serve as a guide to document student performance and progression in the program [15, 16, 30–33].

This study details the integration of EPAs and AS within an OSCE, serving as a transitional step for pharmacy students as they progress towards their APPEs and practical experiences. It further reports on the inclusion of ACPE’s pre-APPE ability statements to help identify essential competencies for APPE-readiness. The study findings reveal a congruence of competencies between EPAs and AS. The 3-station OSCE model used, included 8 EPAs out of 15 EPAs selected by the preceptors who were involved in developing the OSCEs. The method of selecting core EPAs adheres to practices documented in the literature. In this approach, the clinical faculty determines the significance of specific EPAs for pharmacy practice and subsequently incorporate them into the selection process [33]. Furthermore, the activities that featured in each of the OSCE stations are comparable to those used by Bellottie et al. who identified specific activities tailored to pre-APPE core domains as a foundational framework for designing pharmacy practice laboratory exercises [34]. In a recent pilot OSCE test, core competency domains and case scenarios were created through a literature review, brainstorming by researchers, and consensus from external experts, to evaluate its appropriateness as a tool for assessing Korean pharmacy students’ clinical pharmacist competency for APPEs [35].

The incorporation of OSCEs at the end of the P2 year along with the integration of simulation sessions within specific pharmacy courses in P1 and P2 further enhance the preparation of students for their IPPEs in P3. Furthermore, the implementation of the pharmacists’ patient care process (PPCP) model during the P2 plays a pivotal role in promoting students’ readiness for real-world practice scenarios [36]. These strategic program integrations are designed to advance students’ knowledge, and refined clinical skills and professional attitudes such as professional communication, disease screening, medication preparation, dose calculation, and patient education and counseling techniques. While IPPEs are progressively laid out throughout the first 3 professional years (P1, P2, P3), P3 IPPEs are specifically focused on clinical practice that include [480 h], alongside with community and hospital [490 h]. This provides extensive hands-on experiences in various pharmacy practice settings where student performance is assessed via direct preceptor observation.

In this study, students displayed competence in various aspects of AS and EPA. High scores in ethical and legal behavior (AS6) reflected their proficiency in demonstrating related principles. Similarly, a strong average score of 87% across all 3 stations in general communication abilities (AS7) showcased adeptness in effectively communicating with patients and HCPs. Additionally, high evaluation scores in interprofessional collaboration across all stations signify a notable level of competence in working collaboratively with other healthcare team members. The LAU Health Science and Nutrition programs pioneered the implementation of interprofessional education (IPE) in the region, contributing significantly to students’ readiness for interprofessional practice [37]. Nevertheless, consistent low scores in AS6, AS7, and EPA6, particularly noted by healthcare providers (HCP) evaluators in station 3, suggest a need for refining evaluation criteria and additional targeted training.

The implementation of OSCEs may present numerous challenges and is restricted by the availability of resources [38]. From our perspective, the execution of OSCEs has been linked to various challenges concerning faculty involvement as observers or evaluators, particularly in connection with faculty workload and resource constraints. This underscores the importance of developing user-friendly grading rubrics and checklists, and discussing content alignment with course coordinators [19]. In this research, the elevated scores on communication-related statements across all 3 stations validate students’ proficiency in communication skills, achieved through purposeful and focused practice throughout the program. In effect, the observers have mostly likely served as members of the OSCE development team, thereby establishing a robust consensus on the incorporation of EPAs and students’ expectations. This compares to efforts described in the literature about the design and evaluation of a program implemented to prepare clinical faculty members to use EPAs for teaching and assessment in experiential education [39]. Similarly, preceptors’ development programs were reported to be effective in establishing a standardized understanding of the level of entrustability and help ensure that preceptors consistently assign levels of engagement to EPA-based tasks performed by students during their experiential rotations [30]. Another challenge is the limited number of trained faculty in planning and delivering the OSCEs. Institutions should invest in professional development initiatives to train faculty and preceptors on simulation-based education and assessment.

Moreover, the substantial agreement in the students’ evaluations between observers and HCP in station 3, can be attributed to the fact that both are faculty members. However, the observed inconsistency in scores between the observer and the SP evaluator in stations 1 and 2 could be attributed to differences in evaluators’ perspectives and overestimation of student performance by the SP, which suggests a need for better SP training on performance-based assessment. Evidence from a six-year experience suggests that the use of SPs produced accurate simulations and reliable evaluation of student performance [40]. Yet, SPs could not replace pharmacist evaluators in the context of entry-to-practice certification purposes and were actually suggested to potentially rather belong in an educational context [41]. When OSCE scores between faculty and SPs were compared, the SP global assessment scores were significantly higher than faculty scores, suggesting that increased experience within the station and familiarity with the role contribute to enhanced performance [42]. It is important to note that SPs assessed student communication skills. From this perspective, the SPs may have been assessing students’ empathy, patience, caring attitude, whereas the observer was mainly focused on ensuring that required information and questions are covered from a clinical point of view. These results underscore the importance of consistency for a reliable assessment. It may be beneficial to further explore the factors contributing to the discrepancies observed and implement measures to enhance the reliability and validity of the evaluations in all stations. Continuous evaluation and refinement can lead to more accurate assessments of student performance and facilitate targeted interventions for improvement. So far and based on the OSCEs findings and students’ debriefing, curricular enhancements have been implemented in formative simulations (embedded in courses) and in future OSCE stations. For instance, improvement was introduced to the patient education activity in the Dispensing Laboratory course and to the interprofessional communication activity in one of the six courses of the pharmacotherapeutics series.

### Limitations

The authors acknowledge the following study limitations: (1) the design of OSCEs incorporated 3 stations, and to enhance validity, a greater number of stations is recommended; (2) the reported dataset spans a year and it would be valuable to accumulate additional data over a lengthier time to discern more defined patterns in student performance; (3) the performance of students in real-world situations may not be entirely mirrored by their performance on the OSCEs which may question the level or extent of entrustability; (4) Checklists were used to assess students’ performance at each station. However, assessment did not incorporate the evaluators’ assessment of the level of entrustment, indicating an area of improvement.

## Conclusion

The study findings emphasize on the importance of implementing a consistent and reliable process in OSCEs used in assessing the readiness of pharmacy students for Advanced Pharmacy Practice Experiences (APPEs) and real-world practice. It is imperative to explore the contributing factors to observed discrepancies and implement measures that enhance the reliability and validity of evaluations across all stations. The continuous evaluation and refinement process holds the potential to yield more accurate assessments of student performance, enabling targeted interventions for improvement. This study pioneers the application of EPAs and AS as a framework for developing and implementing OSCEs at the P2-P3 level, aiming to assess pharmacy students’ APPE- and practice-readiness. Aligning specific EPAs and AS with learning objectives in various stations, coupled with dedicated evaluation rubrics, has been a key aspect. Despite this progress, ongoing efforts are necessary to enhance the structure, content, and delivery of OSCEs, ensuring broader coverage of EPAs for sustained improvement. Future studies should aim to broaden the scope of OSCE stations to encompass additional EPAs and incorporate levels of entrustment as a scale for evaluation.

### Electronic supplementary material

Below is the link to the electronic supplementary material.

Supplementary Material 1

## Data Availability

All data generated or analyzed during this study are included in this published article. Additional material is available from the corresponding author on reasonable request.

## Abbreviations

AACP: American Association of Colleges of Pharmacy
ACPE: Accreditation Council for Pharmacy Education
APPE: Advanced Pharmacy Practice Experience
AS: Ability Statement
EPA: Entrustable Professional Activity
HCP: Healthcare Professional
IPE: Interprofessional Education
IPPE: Introductory Pharmacy Practice Experience
OSCE: Objective Structured Clinical Examination
P: Pharmacy Professional Year
PPCP: Pharmacists’ Patient Care Process
SP: Standardized Patient
