# Supplementary material for: Entrustable professional activities-based objective structured clinical examinations in a pharmacy curriculum
Source: BMC Med Educ. 2024 Apr 22;24:436. doi: 10.1186/s12909-024-05425-y (PMC11036754; doi:10.1186/s12909-024-05425-y)
Supplement: Supplementary file 1 — Supplementary Material 1 [file 12909_2024_5425_MOESM1_ESM.pdf]

Supplementary Material 1

Patient Education Scenario – OSCE Station 2

Opening Scenario

Mrs C. Barakat is a 50-year-old woman who was admitted to the cardiology ward for Atrial Fibrillation. This morning, you are reviewing her prescription prior to discharge.

You are the student pharmacist caring for Mrs. Barakat. Please take the time to review the patient information and the discharge medication prescribed. Afterwards, enter the patient room to counsel Mrs. Barakat on her anticoagulant medication and address any concerns questions the patient may have.

You have 15 minutes to:

1- (3 minutes) to review and Assess the medication and her anticoagulant information
2- (10 minutes) to complete the discussion with the patient
   a. Open the discussion: Introduction (verify, identify self, reassure patient)
   b. Ask the patient key questions related to her current medication use
   c. Educate the patient about her anticoagulant (i.e. assess, provide info, explain)
   d. Close the discussion
3- (2 minutes) to receive feedback from the evaluator

<u>Patient Profile</u>
Name: Carole Barakat
Age: 50
Gender: Female
Marital status: Married
Height/Weight: 65 Kg, 165 cm
Serum creatinine: 0.7 mg/dL
Socioeconomic status: Stay-at-home mother
Education: college, speaks English and Arabic

Chief complaint: Carole is a 50-year-old stay-at-home mom who was admitted for atrial fibrillation

Relevant past medical history: Hypertension, dyslipidemia
Past surgical history: none
Immunizations are up to date
Allergies: No known drug or food allergies

Medication list:
Telmisartan 80 mg PO daily at bedtime (home med, kept while in the hospital)
Lipitor® (atorvastatin) 20 mg PO once daily (home med, kept while in the hospital)
Xarelto® (rivaroxaban) 20 mg PO once daily
NaCl 0.9% 1 L hydration over 24 hrs (at the hospital)

Relevant social history:
Nonsmoker, drinks on major occasions, denies intravenous drug abuse, marijuana, or illicit drug use.

VS	Temperature	BP	HR	RR	O2 saturation
Date: ________	37°C	115/70 mmHg	68 bpm	15 breath/min	98% on room air

Relevant Family History: Not reported

GA and ROS: all normal

Discharge medications:
Xarelto® (rivaroxaban) 20 mg orally once daily
Telmisartan 80 mg PO at bedtime
Lipitor® (atorvastatin) 20 mg PO once daily
